# Patients With Obesity on the Acute Care Surgery Service: Improving Outcomes

**DOI:** 10.7759/cureus.105729

**Published:** 2026-03-23

**Authors:** Sanjiv F Gray, Beatrice Dieudonne

**Affiliations:** 1 Surgery, University of Central Florida College of Medicine, Orlando, USA; 2 Surgery, Gray’s Clinic, Windermere, USA; 3 Surgery, Gray's Clinic, Windermere, USA

**Keywords:** emergency, nutrition, obesity, surgery, trauma

## Abstract

Obesity is a chronic, progressive disease affecting approximately 40% of adults in the United States and now represents a defining characteristic of the emergency general surgery (EGS) population. Accumulating evidence demonstrates that obesity, particularly class III, class IV, and class V obesity, is associated with significantly worse clinical outcomes following emergency surgical intervention, including higher rates of postoperative complications, venous thromboembolism (VTE), respiratory failure, acute kidney injury, prolonged intensive care unit (ICU) and hospital lengths of stay, increased readmissions, and higher mortality. Importantly, many adverse outcomes are not driven by operative mortality alone but by failure-to-rescue following preventable complications. This narrative review critically examines outcome data for obese EGS patients and argues that systematic adoption of bariatric surgery quality frameworks, including standardized pathways for infection prevention, VTE prophylaxis, nutrition, opioid stewardship, and post-discharge follow-up, represents a pragmatic and evidence-based strategy to improve outcomes in this high-risk population.

## Introduction and background

Emergency general surgery (EGS) services increasingly function at the intersection of acute illness and physiologic stress and chronic disease. Obesity, defined as a body mass index (BMI) ≥ 30 kg/m², is now one of the most prevalent and consequential chronic diseases encountered in EGS practice. Since the 1980s, obesity prevalence has risen steadily, now affecting approximately 40% of US adults and contributing substantially to preventable morbidity and mortality, making it one of the greatest contributors to the burden of chronic disease [1].

More than two decades ago, Brown and Velmahos presciently articulated the emerging reality facing acute care surgeons: “With the combination of the obesity epidemic and the arrival of the acute care surgeon, we must be prepared to care for critically ill obese surgical patients with ever-increasing regularity” [2].

This statement has proven increasingly relevant. Unlike elective surgical populations, EGS patients with obesity present without preoperative optimization, amplifying the physiologic consequences of obesity-related cardiopulmonary, metabolic, renal, and thrombotic dysfunction. As a result, obesity exerts a disproportionate influence on postoperative outcomes, length of stay, readmissions, and failure-to-rescue rates [2-6]. Understanding these outcome patterns and adopting systems proven effective in bariatric surgery are essential to improving emergency surgical care [7-10]. Systems preparedness for outcome improvement: “An increased recognition of obesity as a chronic disease and a better understanding of its pathophysiology can allow for proper preparation and accommodative measures to improve resuscitation and subsequent care, thereby improving trauma outcomes” [11]. This narrative review examines the patterns of morbidity and risk factors for mortality among patients with obesity undergoing EGS and provides practical recommendations to optimize clinical management.

## Review

Literature search

A structured literature search was conducted to identify studies addressing outcomes and management strategies in patients with obesity undergoing EGS. Electronic databases, including PubMed/MEDLINE and Google Scholar, were searched for articles published between 2000 and 2024. Search terms included combinations of obesity, morbid obesity, EGS, acute care surgery, venous thromboembolism (VTE), respiratory failure, acute kidney injury, nutrition, and sarcopenic obesity. Studies were included if they evaluated adult patients with obesity undergoing emergency or urgent surgical procedures and reported perioperative outcomes or management considerations relevant to acute care surgery. Additional relevant articles were identified through manual review of reference lists and relevant clinical guidelines. Findings were synthesized narratively to highlight patterns of morbidity, risk factors for mortality, and practical strategies to optimize care in obese EGS patients.

Outcomes of obese patients in EGS

While early trauma and surgical studies produced inconsistent conclusions regarding obesity and mortality, contemporary data demonstrate a clear, BMI-dependent relationship between obesity severity and adverse outcomes in emergency surgical populations [3-6,12]. Large NSQIP (National Surgical Quality Improvement Program) analyses show that patients with class III obesity (BMI ≥ 30 kg/m²), class IV obesity (BMI ≥ 40 kg/m²), and class V obesity (BMI ≥ 50 kg/m²) experience significantly higher 30-day mortality following emergency abdominal surgery compared with non-obese patients [3-6]. Mortality rates rise progressively with BMI class, reaching over 7% in super-super-obese patients. Importantly, obesity-related mortality in EGS is often mediated through failure-to-rescue, rather than intraoperative death. Obese patients are more likely to develop postoperative complications, particularly infection, respiratory failure, renal injury, and VTE, and are less likely to recover once complications occur [2-6]. Failure-to-rescue refers to death following the development of a postoperative complication and reflects a healthcare system’s ability to recognize and effectively manage complications once they occur. Clinicians with knowledge of the complication patterns will be more prepared to respond.

Multiple large database studies consistently demonstrate that patients with obesity undergoing EGS experience significantly higher complication rates compared to their non-obese counterparts. These patients have increased rates of surgical site infection and wound complications, as well as a higher incidence of VTE, including deep vein thrombosis and pulmonary embolism. Respiratory complications, including postoperative respiratory failure and the need for reintubation, occur more frequently in this population. Obese EGS patients are also at increased risk of acute kidney injury and may require renal replacement therapy more often. Collectively, these complications contribute to longer intensive care unit (ICU) and overall hospital lengths of stay, as well as higher rates of unplanned readmission, particularly among patients with severe obesity.

Obesity-related outcomes are driven by converging physiologic and system-level factors. Obesity alters respiratory mechanics, cardiovascular reserve, renal perfusion, coagulation pathways, immune function, and pharmacokinetics [2,3]. These physiologic changes predispose patients to complications that are particularly consequential in emergency surgery. At the systems level, obese patients are more likely to experience delays in diagnosis due to limited physical examination and imaging challenges, suboptimal medication dosing, delayed mobilization, inadequate VTE prophylaxis [5,6,12,13], and delayed recognition of postoperative deterioration. Collectively, these factors increase complication burden and reduce rescue capacity.

Patients with obesity presenting to EGS carry a substantial burden of chronic disease that significantly influences perioperative risk and outcomes. Common obesity-related comorbidities include obstructive sleep apnea and obesity hypoventilation syndrome, cardiopulmonary disease, diabetes mellitus, chronic kidney disease, metabolic dysfunction, steatotic liver disease, venous stasis disease, and malignancy. These conditions frequently coexist, reducing physiologic reserve and tolerance to acute surgical stress. As a result, obese EGS patients are more likely to be classified as ASA (American Society of Anaesthesiologists) III-IV, a designation consistently associated with increased postoperative complications, prolonged ICU and hospital length of stay, and higher mortality. Elevated ASA status reflects not only baseline disease severity but also diminished rescue capacity once complications occur, contributing to higher failure-to-rescue rates in this population. Moreover, in the emergency setting, obesity-related comorbidities are often undiagnosed or poorly controlled, further amplifying the risk [2-4]. Recognition of elevated ASA classification in obese EGS patients should prompt heightened perioperative vigilance, early ICU consideration, and proactive implementation of obesity-specific mitigation strategies, including respiratory support, renal protection, weight-based VTE prophylaxis, and early nutrition optimization [2,5,6,10,14,15].

The case for bariatric quality frameworks in EGS

Bariatric surgery programs have confronted obesity-related risk directly for decades. Through the Metabolic and Bariatric Surgery Accreditation and Quality Improvement Program (MBSAQIP) and national quality collaboratives, bariatric centers have developed structured, reproducible pathways that reduce complications despite operating on one of the highest-risk surgical populations.

EGS, by contrast, often lacks standardized obesity-specific pathways. This discrepancy represents an opportunity for performance improvement. Many of the adverse outcomes observed in obese EGS patients mirror those historically seen in bariatric surgery prior to widespread quality standardization [7-10].

Three bariatric quality initiatives, such as Decreasing Readmissions through Opportunities Provided (DROP), Employing New Enhanced Recovery Goals for Bariatric Surgery (ENERGY), and Bariatric Surgery Targeting Opioid Prescriptions (BSTOP), are particularly relevant to EGS and directly address the complications that drive poor outcomes in obese emergency surgery patients [7-10]. The DROP initiative demonstrated that structured discharge education, follow-up phone calls, and early outpatient engagement significantly reduced 30-day readmissions in bariatric surgery. In EGS, obese patients experience disproportionately high readmission rates. Applying DROP principles through standardized discharge education, early post-discharge contact, and clear escalation pathways addresses a major driver of poor outcomes. Postoperative infection is one of the most common complications in obese EGS patients and a major contributor to failure-to-rescue. The ENERGY initiative demonstrated that infection reduction requires system-level intervention, not isolated surgeon behavior [8]. The core principles applicable to EGS include weight-appropriate antibiotic selection and dosing, strict adherence to prophylaxis protocols, perioperative glucose control, and early nutrition optimization. Finally, opioid-related respiratory depression disproportionately affects obese patients, particularly those with obstructive sleep apnea. The BSTOP initiative demonstrated that multimodal analgesia significantly reduces opioid exposure without compromising pain control [9]. Adopting BSTOP principles in EGS by using non-steroidal anti-inflammatory drugs (NSAIDs), acetaminophen, regional anesthesia, and non-pharmacologic adjuncts may reduce respiratory failure, reintubation, and prolonged ICU stays.

Venous thromboembolism in obese EGS patients

Venous thromboembolism (VTE) is one of the most frequent, morbid, and preventable complications in obese EGS patients [2,5,6,12,13,16]. Obesity is an established prothrombotic condition characterized by elevated platelet counts, increased levels of clotting factors, particularly factor IX, reduced fibrinolysis, lower D-dimer levels, chronic low-grade inflammation, and endothelial dysfunction.

Prospective trauma data demonstrate that for every 5 kg/m² increase in BMI, the odds of thromboembolic complications increase by approximately 85% [12]. This hypercoagulable state is further amplified in emergency surgical patients by acute inflammation, immobility, tissue injury, and sepsis. Large ACS-NSQIP analyses involving tens of thousands of EGS patients demonstrate a clear, stepwise increase in deep venous thrombosis and pulmonary embolism rates with increasing BMI [6,13]. Importantly, VTE in this population is not a benign complication, as post-VTE mortality exceeds 11%, underscoring its role as a major contributor to failure-to-rescue [16]. The high mortality is likely due to delayed diagnosis, pre-existing conditions, and the reduced physiologic reserve secondary to the EGS. Pulmonary embolism remains a leading cause of preventable postoperative death in obese emergency surgery patients [2,6,12,13,16]. Standard fixed-dose pharmacologic prophylaxis is frequently inadequate in obese patients due to altered drug pharmacokinetics and increased volume of distribution [5,6,17-19]. Multiple studies demonstrate subtherapeutic anti-Xa levels when standard low-molecular-weight heparin (LMWH) dosing is used in patients with BMI ≥ 35-40 kg/m² [17-19]. Emergency surgery further compounds this issue, as prophylaxis is often delayed or interrupted due to bleeding risk, perioperative holding, invasive procedures, or, often, simply not being ordered [5,6,13].

Evidence from bariatric and medically ill obese populations supports weight-based dosing of LMWH to achieve therapeutic prophylactic anti-Xa levels without increasing bleeding risk [17-19]. Weight-based enoxaparin 0.5 mg/kg once daily has been shown to achieve therapeutic anti-Xa levels in patients with BMI ≥ 40 kg/m² (Table 1) [19]. Furthermore, anti-Xa monitoring should be considered for high-risk patients. A critical insight from bariatric surgery literature is that many VTE events occur after discharge, particularly in high-risk patients [16,20]. Emergency surgery patients with obesity share many of the same risk factors of immobility, inflammation, and delayed recovery, but rarely receive extended prophylaxis [5,6,13,16,20]. Risk stratification tools and bariatric-derived protocols may support extended prophylaxis in patients with BMI ≥ 40 kg/m², prior VTE, prolonged hospitalization, reduced mobility, active malignancy or inflammatory conditions, and those who require reoperation. Direct oral anticoagulants may play a role in extended prophylaxis, although data in post-EGS populations remain limited [16,20]. Routine prophylactic placement of inferior vena cava filters has not been shown to prevent pulmonary embolism and is not recommended [2,16]. Inferior vena cava (IVC) filters should be reserved for select high-risk patients with documented acute deep vein thrombosis (DVT) and contraindications to anticoagulation. Their use should be deliberate, time-limited, and coupled with a clear retrieval plan. Implementing bariatric-derived VTE protocols represents one of the most actionable, evidence-based opportunities to reduce preventable death in obese EGS patients [5,6,13,16,20].

**Table 1:** Patients who may benefit from extended VTE prophylaxis after emergency general surgery VTE: Venous thromboembolism.

BMI ≥ 40 kg/m²
Prior VTE
Prolonged hospitalization
Reduced mobility
Active malignancy
Inflammatory conditions
Reoperation

Initial resuscitation of the obese EGS patients

Initial resuscitation of patients with obesity presenting to EGS requires anticipation of obesity-specific anatomic and physiologic challenges that directly affect airway management, ventilation, and hemodynamic assessment. Failure to recognize and proactively address these factors contributes to early complications and downstream failure-to-rescue [2,11].

Airway

**Figure 1:**
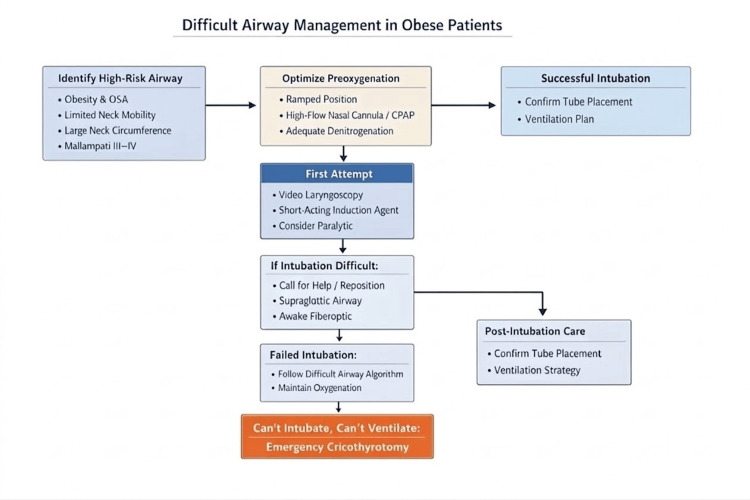
Airway management for the patient with obesity OSA: Obstructive sleep apnea; CPAP: Continuous positive airway pressure. Image credit: This image was created by the authors using MS PowerPoint (Microsoft Corp., Redmond, WA).

Obesity and obstructive sleep apnea are independent predictors of difficult airway management. Excess soft tissue, limited neck mobility, reduced functional residual capacity (FRC), and supine positioning result in rapid oxygen desaturation during apnea, often occurring in less than one minute. End-expiratory lung volume may decrease by nearly 70% following induction in the supine position. Clinicians should be aware of their institution's difficult airway algorithm, as airway management should be planned rather than reactive. Key principles include aggressive preoxygenation, head-elevated or reverse Trendelenburg positioning, early use of video laryngoscopy, and readiness for difficult airway adjuncts (Figure 1). Bag-mask ventilation should not be delayed if oxygenation is inadequate. High-flow nasal cannula oxygen may extend safe apnea time and facilitate apneic oxygenation. Given the high risk of post-intubation hypoventilation, extubation planning should begin at the time of intubation [2,11].

Breathing

Ventilatory management must account for altered respiratory mechanics in obesity. Reduced chest wall compliance, elevated intra-abdominal pressure, and increased oxygen consumption predispose patients to atelectasis, hypoxemia, and ventilator-associated lung injury. Lung-protective ventilation using tidal volumes based on ideal body weight, rather than actual body weight, is essential.

Positive end-expiratory pressure should be applied early to prevent alveolar collapse, and recruitment maneuvers may be used judiciously. Post-resuscitation, obese patients are at high risk for respiratory failure, particularly in the setting of opioid administration. Early application of continuous positive airway pressure or noninvasive ventilation should be strongly considered, even in patients without a formal diagnosis of obstructive sleep apnea, with end-tidal CO₂ monitoring when available [2,9,11,14].

Circulation

Circulatory assessment in obese patients is challenging and frequently inaccurate. Noninvasive blood pressure measurements may be unreliable due to inappropriate cuff sizing. Obesity is associated with increased circulating blood volume, elevated cardiac output, ventricular hypertrophy, and endothelial dysfunction, complicating the interpretation of traditional resuscitation endpoints.

**Figure 2:**
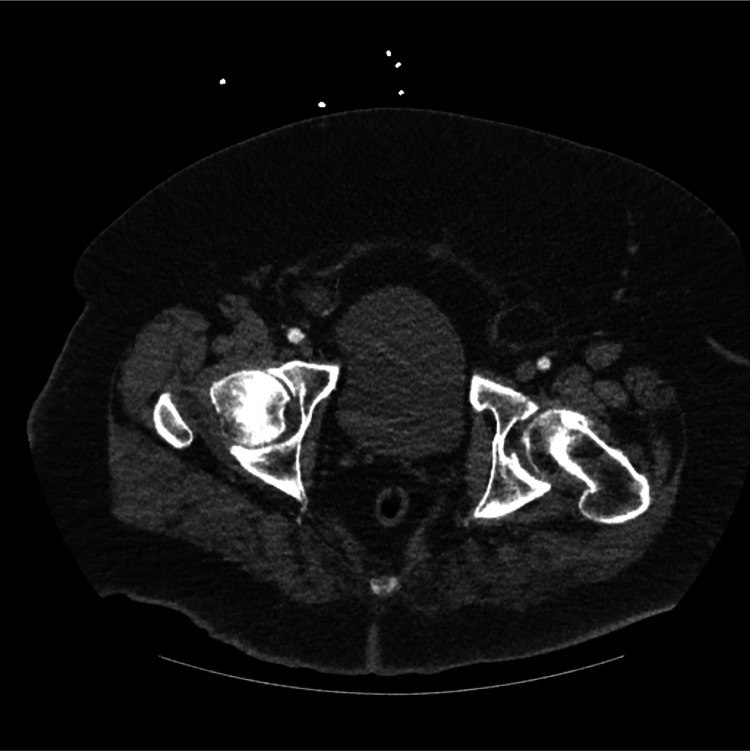
CT scan image demonstrating femoral vessels at 13 cm depth, illustrating challenges of vascular access in severe obesity Image credit: Author’s original image. Used with patient consent.

**Figure 3:**
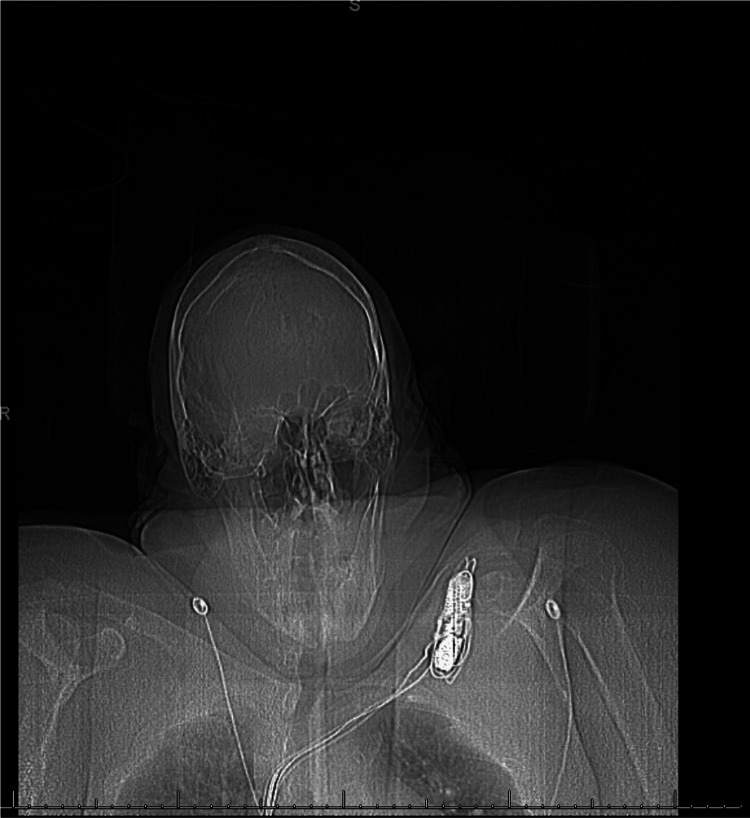
Anticipated challenging central venous access due to body habitus Image credit: Author’s original image. Used with patient consent.

Resuscitation should emphasize goal-directed strategies, using dynamic measures of perfusion when available, early lactate assessment and clearance, and close monitoring of urine output trends. Both under-resuscitation and over-resuscitation are harmful; excessive fluid administration increases the risk of renal congestion, abdominal compartment syndrome, and respiratory compromise, while inadequate resuscitation exacerbates shock and acute kidney injury. Early arterial and central venous access should be considered when noninvasive monitoring is unreliable [2,9,11]. Figures 2, 3 show examples of anticipated difficult vascular access due to anatomical reasons.

EGS conditions and obesity-specific considerations

Complicated Diverticulitis

Obesity is associated with higher rates of complicated diverticulitis, including abscess formation and perforation. Percutaneous drainage and nonoperative management may be technically challenging. When operative intervention is required, laparoscopy should be considered when feasible, with efforts to avoid stoma creation when possible due to higher stoma-related morbidity in obese patients. There may be a role for laparoscopic lavage as the technique is associated with a lower stoma rate despite more recurrent episodes [2,3]. If an ostomy is required, it should be placed in the upper abdomen, where the abdominal wall has less adipose tissue.

Appendicitis

Patients with obesity frequently present later in the disease course, with higher rates of perforation and abscess [2,3]. Diagnostic imaging may be delayed or equivocal. Laparoscopic appendectomy remains preferred but may require altered port placement and additional instrumentation to aid in exposure. This may include a trocar in the right upper abdomen to aid with retraction.

Open Abdomen Management

Obese patients undergoing damage-control laparotomy are at increased risk for prolonged open abdomen duration, fascial closure failure, wound complications, and ventral hernia formation. Early nutritional support and timely closure strategies are critical [10]. Early application of traction and sequential closure techniques should be considered to aid in timely closure. Placing traction on the linea alba and Scarpa's fascia may facilitate closure over time.

Necrotizing Soft Tissue Infection

Obesity is a major risk factor for necrotizing soft tissue infection. Diagnosis may be delayed due to impaired physical examination. These patients often require larger debridement fields, multiple operations, and prolonged ICU care, contributing to high morbidity and mortality. Expertise in wound care and nutrition is crucial for success.

Post-Bariatric Complications

Small bowel obstruction and internal hernia following bariatric surgery are life-threatening emergencies. Computed tomography may fail to diagnose internal hernias in up to 66% of cases [21]. Clinical suspicion should prompt early diagnostic laparoscopy despite equivocal imaging to prevent catastrophic small bowel ischemia and subsequent risk for short bowel syndrome and malnutrition [2,10]. Small bowel obstruction in patients with prior bariatric surgery involving anastomoses is more likely to require surgical intervention since the etiology is different. Early laparoscopy is recommended to prevent complications. Advanced laparoscopy skills and knowledge of bariatric procedures are required. Caution is advised with placement of a nasogastric tube, as iatrogenic perforation has been reported. In addition, the nasogastric tube is often ineffective for decompression after a gastric bypass or duodenal switch.

Marginal and Duodenal Ulcers

Marginal ulceration and duodenal ulcer may present with bleeding or perforation. The incidence of peptic ulcer disease increases with obesity. Management includes cessation of offending agents such as smoking and NSAIDs, acid suppression, and operative intervention when indicated, with attention to altered anatomy. Minimal invasive techniques may reduce wound complications. The ulcer can be managed with primary repair, omental patch, or revisional surgery.

Biliary Disease

Cholecystectomy in obese patients is complicated by hepatomegaly and fatty liver disease, increasing technical difficulty and bleeding risk. However, studies show no increased risk of complications overall [2,3]. We recommend using a secured footboard to facilitate steep positioning and ensure the patient is properly secured to the operating room table. Choledocholithiasis in post-bariatric patients may require advanced endoscopic or surgical techniques. Endoscopic ultrasound-directed transgastric endoscopic retrograde cholangiopancreatography (ERCP), abbreviated as EDGE, is emerging as an option for ERCP post-gastric bypass. Laparoscopic gastrotomy-assisted ERCP has a similar success rate [2].

Acute kidney injury, renal failure, and rhabdomyolysis in obese EGS patients

Acute kidney injury (AKI) is a common and clinically significant complication in patients with obesity undergoing EGS and is a major contributor to adverse outcomes. Obesity is independently associated with chronic kidney disease and reduced renal reserve, increasing susceptibility to AKI during acute illness, sepsis, hemorrhage, and operative stress. Obesity-related renal vulnerability is multifactorial. Increased intra-abdominal pressure from central adiposity reduces renal perfusion and venous outflow, while chronic hyperfiltration and glomerulomegaly limit the kidney’s ability to tolerate acute insults [2,14]. Systemic inflammation, endothelial dysfunction, insulin resistance, and oxidative stress further impair renal autoregulation. In the emergency surgical setting, these chronic alterations are compounded by hypotension, vasopressor exposure, contrast administration, and sepsis.

Rhabdomyolysis is a renal risk amplifier. Patients with obesity are at increased risk for rhabdomyolysis in the emergency surgery and critical care setting [15]. Contributing factors include prolonged immobilization, compartment syndromes, ischemia-reperfusion injury, pressure-related muscle injury during lengthy operations, and severe infections. Clinical assessment should include a high index of suspicion in patients with unexplained AKI, dark urine, rising creatinine kinase levels, or disproportionate renal dysfunction relative to hemodynamic instability. Laboratory evaluation should include serial creatine kinase measurements, renal function testing, electrolyte monitoring, myoglobin, and urine analysis for heme-positive, red blood cell-negative findings. Management centers on early recognition, aggressive but judicious fluid resuscitation to maintain renal perfusion, correction of electrolyte abnormalities, and avoidance of additional nephrotoxins. Volume management must be individualized, as over-resuscitation increases the risk of renal congestion and abdominal compartment syndrome, while under-resuscitation exacerbates myoglobin-induced renal injury [2,14,15].

Accurate volume assessment in obese EGS patients remains challenging, as physical examination and static preload measures are often unreliable. Goal-directed resuscitation strategies, close urine output monitoring, and early multidisciplinary involvement are essential. Medication dosing should be individualized, with prompt adjustment of renally cleared agents. Renal failure and rhabdomyolysis frequently coexist with other obesity-associated complications, including respiratory failure, VTE, and infection, and serve as both markers and mediators of multisystem organ dysfunction. Incorporating renal risk stratification, rhabdomyolysis surveillance, and renal-protective strategies into obesity-specific EGS pathways represents an important opportunity to reduce AKI incidence and improve outcomes [2,14,15].

Nutrition optimization in obese EGS patients

Despite excess adiposity, patients with obesity presenting to EGS are frequently malnourished, a condition increasingly recognized as sarcopenic obesity, in which excess fat mass coexists with reduced skeletal muscle mass and function [10]. Sarcopenic obesity is particularly prevalent in critically ill and post-bariatric patients and is strongly associated with poor wound healing, immune dysfunction, respiratory failure, prolonged length of stay, and increased mortality. Early and structured nutritional risk assessment is essential in obese EGS patients. The Global Leadership Initiative on Malnutrition (GLIM) criteria, used in conjunction with American Society for Parenteral and Enteral Nutrition (ASPEN) guidelines, provides a robust framework for identifying malnutrition in this population. GLIM integrates phenotypic criteria (including reduced muscle mass) with etiologic criteria (reduced intake, impaired absorption, inflammation, or disease burden), allowing accurate diagnosis even in patients with elevated BMI [22].

Importantly, reliance on BMI alone masks malnutrition in patients with sarcopenic obesity. The GLIM framework differs from traditional malnutrition assessment by incorporating objective assessment of skeletal muscle mass as a diagnostic criterion. Traditional approaches often rely on weight loss or low BMI, which may fail to detect malnutrition in patients with obesity. By including reduced muscle mass, GLIM improves identification of sarcopenic obesity, a common and clinically significant condition in acute care surgery patients. Assessment of muscle mass should therefore be incorporated into routine evaluation. Computed tomography (CT) imaging, frequently obtained for diagnostic purposes in EGS patients, can be leveraged to assess skeletal muscle area and quality (e.g., at the L3 vertebral level). This may require specialized software or training. Bedside ultrasound provides an additional, radiation-free modality to evaluate muscle thickness and quality, particularly in the ICU, and may be useful for serial assessment when CT is unavailable or impractical [10,22]. While not yet standard practice, incorporating CT-based sarcopenia assessment into clinical workflows represents an opportunity for future quality improvement.

Consistent with ASPEN recommendations, hypocaloric, high-protein nutrition is the preferred strategy for critically ill patients with obesity [10]. This approach minimizes overfeeding while preserving lean body mass, which is particularly critical in patients with sarcopenic obesity. Energy requirements should be estimated using validated predictive equations rather than weight-based formulas, while protein provision should be prioritized to attenuate catabolism. Early enteral nutrition remains the preferred route whenever feasible, supporting gut integrity, immune function, and glycemic control. These 2013 guidelines remain current and represent an area for further research. Parenteral nutrition should be reserved for patients who cannot tolerate or achieve adequate enteral intake despite optimization. Patients on parenteral nutrition require increased vigilance to prevent infection and may require insulin for glucose management.

Patients with prior bariatric surgery represent a high-risk subgroup for both macronutrient and micronutrient deficiencies. Common deficiencies include iron, vitamin B12, folate, thiamine, vitamin D, zinc, copper, and selenium [10]. Acute illness may rapidly exacerbate these deficits, and empiric supplementation should be considered in high-risk patients while awaiting laboratory confirmation. Early empiric thiamine administration is crucial for the prevention of long-term morbidity. The recommended thiamine dose ranges from 200 to 500 mg, depending on the clinical context. Patients with suspected Wernicke's encephalopathy should receive the high dose for three to five days.

**Figure 4:**
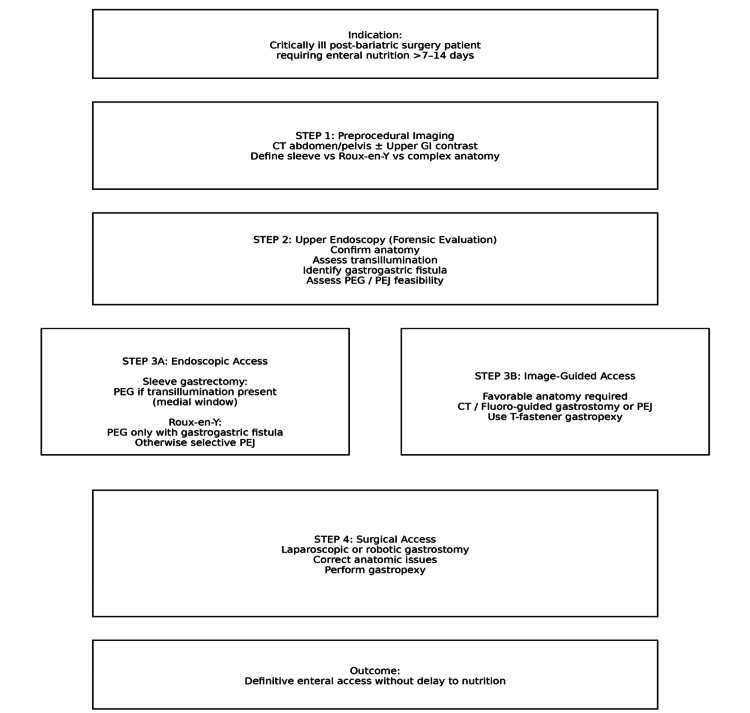
Gray-Swedberg-Hall-McCoy algorithm for feeding access in the post-bariatric surgery patient Presented at ASPEN 2026 Meeting, Long Beach, California. PEG: Percutaneous endoscopic gastrostomy; PEJ: Percutaneous endoscopic jejunostomy. Image credit: This image was created by the authors using MS PowerPoint (Microsoft Corp., Redmond, WA).

Feeding access in post-bariatric surgery patients requires careful planning due to altered anatomy. Nasogastric access may be ineffective or contraindicated depending on the surgical procedure performed. In patients with Roux-en-Y gastric bypass, enteral access may require placement of a jejunostomy tube, gastrostomy into the excluded stomach, or fluoroscopic or endoscopic-guided access in collaboration with gastroenterology or interventional radiology (Figure 4). Early multidisciplinary coordination is essential to avoid prolonged undernutrition.

Nutrition optimization should be considered a core component of resuscitation in obese EGS patients rather than an adjunctive therapy. Incorporation of GLIM-based screening, routine assessment for sarcopenic obesity using CT or ultrasound, ASPEN-guided hypocaloric high-protein feeding strategies, and proactive planning for post-bariatric feeding access into standardized EGS pathways has the potential to reduce infectious complications, improve wound healing, shorten length of stay, and enhance rescue capacity [8,10,21].

## Conclusions

Obesity is one of the most powerful determinants of outcome in EGS. Contemporary evidence consistently demonstrates that patients with obesity experience higher complication rates, longer hospital length of stay, increased readmissions, and higher mortality, often driven by failure-to-rescue rather than operative death. Bariatric surgery programs have already demonstrated that these risks can be mitigated through structured, evidence-based quality frameworks. EGS must now adopt similar system-level approaches. Leaders in EGS, hospital administrators, and quality improvement committees should prioritize the development and implementation of obesity-focused clinical pathways that address infection prevention, weight-appropriate VTE prophylaxis, opioid stewardship, nutrition optimization, and structured post-discharge follow-up.

Failure to systematically address obesity within EGS pathways will perpetuate preventable morbidity and mortality in a rapidly growing patient population. Conversely, integrating obesity-specific quality improvement strategies into emergency surgical care represents an immediate and actionable opportunity to reduce complications, improve rescue capacity, and meaningfully improve outcomes for patients with obesity requiring EGS. The obesity epidemic has already transformed the EGS population; the next step is ensuring that emergency surgical systems evolve to meet it.
